# Genetic Variation in Plant CYP51s Confers Resistance against Voriconazole, a Novel Inhibitor of Brassinosteroid-Dependent Sterol Biosynthesis

**DOI:** 10.1371/journal.pone.0053650

**Published:** 2013-01-15

**Authors:** Wilfried Rozhon, Sigrid Husar, Florian Kalaivanan, Mamoona Khan, Markus Idlhammer, Daria Shumilina, Theo Lange, Thomas Hoffmann, Wilfried Schwab, Shozo Fujioka, Brigitte Poppenberger

**Affiliations:** 1 Biotechnology of Horticultural Crops, Center for Life and Food Sciences Weihenstephan, Technische Universität München, Freising, Germany; 2 Max F. Perutz Laboratories, University of Vienna, Vienna, Austria; 3 Institue of Plant Biology, Technical University of Braunschweig, Braunschweig, Germany; 4 Biotechnology of Natural Products, Center for Life and Food Sciences Weihenstephan, Technische Universität München, Freising, Germany; 5 RIKEN Advanced Science Institute, Wako-shi, Saitama, Japan; Wake Forest University, United States of America

## Abstract

Brassinosteroids (BRs) are plant steroid hormones with structural similarity to mammalian sex steroids and ecdysteroids from insects. The BRs are synthesized from sterols and are essential regulators of cell division, cell elongation and cell differentiation. In this work we show that voriconazole, an antifungal therapeutic drug used in human and veterinary medicine, severely impairs plant growth by inhibiting sterol-14α-demethylation and thereby interfering with BR production. The plant growth regulatory properties of voriconazole and related triazoles were identified in a screen for compounds with the ability to alter BR homeostasis. Voriconazole suppressed growth of the model plant *Arabidopsis thaliana* and of a wide range of both monocotyledonous and dicotyledonous plants. We uncover that voriconazole toxicity in plants is a result of a deficiency in BRs that stems from an inhibition of the cytochrome P450 CYP51, which catalyzes a step of BR-dependent sterol biosynthesis. Interestingly, we found that the woodland strawberry *Fragaria vesca,* a member of the *Rosaceae*, is naturally voriconazole resistant and that this resistance is conferred by the specific CYP51 variant of *F. vesca*. The potential of voriconazole as a novel tool for plant research is discussed.

## Introduction

Higher plants synthesize a complex mixture of small molecular weight compounds, which act as signaling molecules in low quantities to regulate growth and development. One group of plant growth regulatory substances is the brassinosteroids (BRs), steroid hormones similar in their structure to steroid hormones of mammals and ecdysteroids of insects [1]. The BRs regulate cell elongation, cell division and cell differentiation and thereby coordinate developmental programs leading to morphogenesis [2], [3].

BRs are synthesized from campesterol a bulk sterol formed in the so-called BR-dependent branch of general sterol synthesis. Mutants affected in genes acting in this branch such as *dwf7/ste1*, *dwf5* or *dwf1/dim* are characterized by similar phenotypes as BR-deficient plants: severe dwarfism, reduced cell elongation, reduced male fertility, delayed flowering and senescence. Importantly these mutants are rescued by BR application; thus, the growth defects of these plants are predominantly caused by BR-deficiency. Mutants blocked in earlier steps of sterol synthesis e.g. *smt1*, *cyp51A2*, *fk/hyd2*, or *hyd1* show additional phenotypes including aberrant embryogenesis and seed development and are not rescued by BR application, which has been suggested to indicate that sterols also regulate plant development by BR-independent means [4], [5].

The biosynthetic end product of BR biosynthesis is brassinolide (BL). BL is the biologically most active BR in many plant species and acts at minute concentrations in the pM to nM range [6]. Even small changes in bioactive BR levels lead to severe growth defects. Thus, plants have evolved multiple control mechanisms for regulating BR homeostasis including the inactivation of the hormones by catabolism [7], [8] as well as a feedback regulation of BR biosynthesis by BL from the signaling pathway [9]. BRs are perceived by a BRI1 and BAK1 containing receptor kinase complex, which triggers a phosphorylation-dependent signal transduction cascade that ultimately leads to de-phosphorylation and activation of the BES1/BZR1 family of transcription factors [10], [11], [12], which, together with different types of bHLH transcription factors [13], [14], [15], [16], control BR target gene expression.

**Figure 1:**
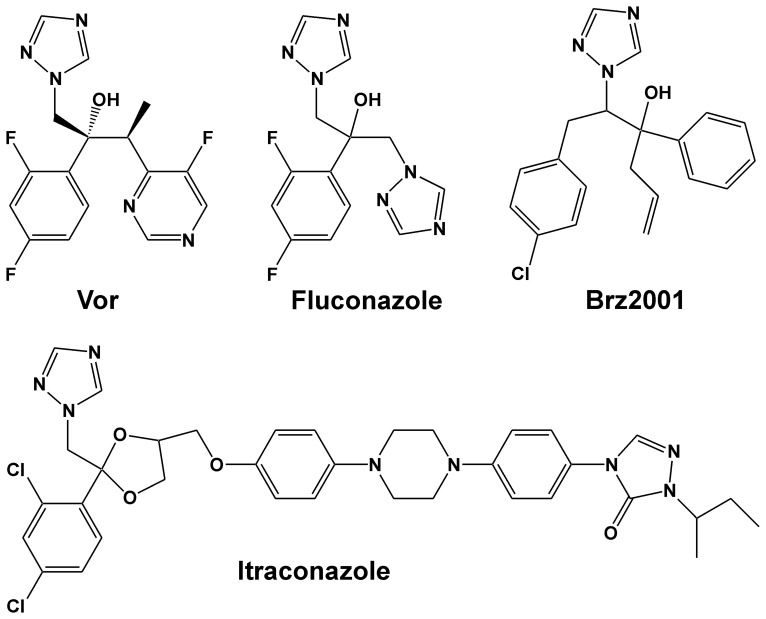
Structures of the triazoles voriconazole, fluconazole and itraconazole and the BR biosynthesis inhibitor Brz2001.

Although BRs were discovered in the 1970s only, the biosynthesis, signal transduction and functions of BRs are well characterized today. This rapid progress has been made possible by the application of multiple strategies for elucidating BR action including forward genetic approaches facilitated by the use of BR biosynthesis inhibitors [17]. The use of chemical inhibitors of enzyme function is a powerful tool to alter metabolic pathways or signal transduction cascades in cellular organisms. Their most prominent applications are as pharmaceuticals for the treatment of diseases and as pesticides and herbicides in agriculture. In recent years chemical inhibitors have also become invaluable tools for research, applied in ‘chemical biology’ to the study and manipulation of biological systems [18], [19], [20]. Chemical inhibitors, which target BR biosynthesis known to date are brassinazole (Brz) [21], [22], Brz2001 [23] (Figure 1), Brz220 [24] and propiconazole [24], [25]. So far only the molecular targets of Brz and Brz220 have been identified. Both triazoles inhibit the activity of the cytochrome P450 DWF4, an enzyme that catalyzes a rate-limiting step of BR biosynthesis, by binding to its prosthetic haem group [26], [27]. Sterol biosynthesis inhibitors active in plants have also been characterized although their modes of action have remained largely elusive [28], [29], [30]. They include compounds such as the herbicide LAB 170250F, which impairs sterol synthesis by acting on cytochrome P450s that catalyze obtusifoliol-14-demethylation [28], [29], [31], [30].

In this study we identify voriconazole and related triazoles, used as antifungal therapeutic drugs for the treatment of *Aspergillus sp*. and *Candida sp.* infections [32] as potent inhibitors of BR-dependant sterol biosynthesis in plants. Voriconazole acts at µM concentrations, is incorporated by plants within a few hours, decreases sterol and BR contents and severely impairs growth of both monocotyledonous and dicotyledonous plant species, with one notable exception: the woodland strawberry *Fragaria vesca*. *F*. *vesca* was employed as a model to elucidate modes of voriconazole toxicity in plants.

## Results

### Voriconazole Induces Phenotypes Indicative of BR Deficiency in Arabidopsis and Cress

**Figure 2:**
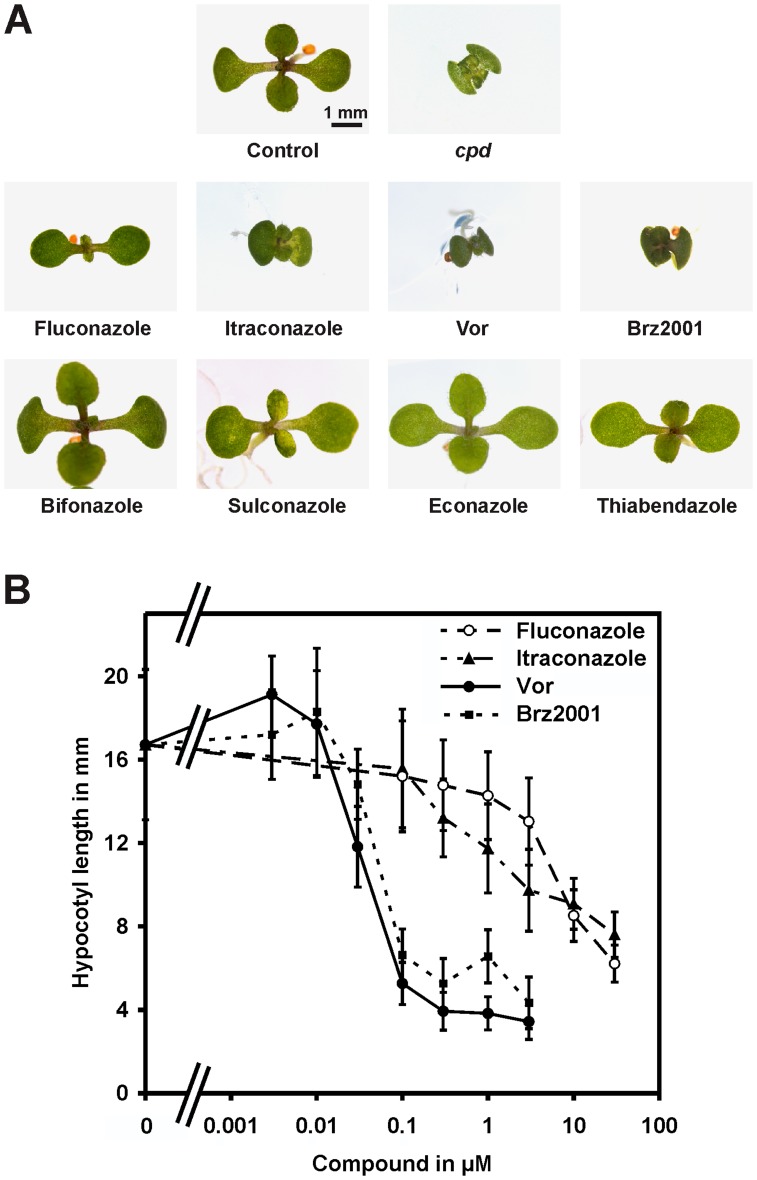
Voriconazole, itraconazole and fluconazole induce phenotypes indicative of BR deficiency in arabidopsis and cress. (**A**) Arabidopsis plants grown on plates containing 5 µM of the indicated compounds under long-day conditions for 7 days. (**B**) Hypocotyl length of cress seedlings grown for 3 days on plates containing different concentrations of the indicated compounds. Data are the mean ± SD of 30 seedlings measured.

In an approach to assess the ability of pharmaceuticals to alter BR homeostasis of plants we found that fluconazole, a triazole used as an antifungal therapeutic drug, induced phenotypes indicative of BR deficiency in *Arabidopsis thaliana* (arabidopsis). Arabidopsis plants grown in ATS media supplemented with 25 µM of fluconazole were characterized by a reduced overall size, shortened hypocotyls and dark-green, epinastic leaves (Figure S1) resembling BR-deficient mutants such as *cpd* [33] or *det2-1* [34], as well as plants treated with known BR biosynthesis inhibitors such as Brz2001 [23]. An analysis of structurally related compounds (Figure 1) revealed that application of itraconazole and particularly voriconazole caused identical phenotypes, whereas imidazole derivatives including bifonazole, econazole, sulconazole and thiabendazole did not induce such effects (Figure 2A, 2B).

To compare the potency of the identified compounds in affecting plant growth we decided to analyze their ability to inhibit hypocotyl elongation of cress, an assay frequently used to investigate BR action [21], [22], [35], [24], [36]. As shown in Figure 2B, voriconazole strongly reduced hypocotyl elongation of cress with significant effects detectable at a concentration of 30 nM. Fluconazole and itraconazole similarly inhibited cress hypocotyl growth, but higher amounts were necessary to cause similar phenotypic responses (Figure 2B).

BRs are essential regulators of cell elongation. Thus, BR-deficiency is evidenced at a phenotypic level by reduced cell elongation rates [37], [33], [38]. To investigate if the phenotypes induced by voriconazole application were also characterized by impaired cell elongation epidermal hypocotyl cells of voriconazole treated cress and zinnia seedlings were visualized by electron microscopy. The results are documented in Figure S2 and show that the cell length of treated plants was clearly reduced as compared to untreated controls.

Therefore an external application of fluconazole, itraconazole and particularly voriconazole induces severe growth defects indicative of BR deficiency in arabidopsis and cress.

### Voriconazole Application Inhibits Sterol Biosynthesis and Impairs BR Biosynthesis

Several triazoles such as Brz, uniconazole, paclobutrazol, triadimefon and LAB170250F are known to inhibit plant growth. However, their activities vary as they interfere with the biosynthesis of different metabolites important for plant development including BRs, gibberellins (GAs) and sterols, respectively [28], [39], [21], [22], [24], [36].

**Figure 3:**
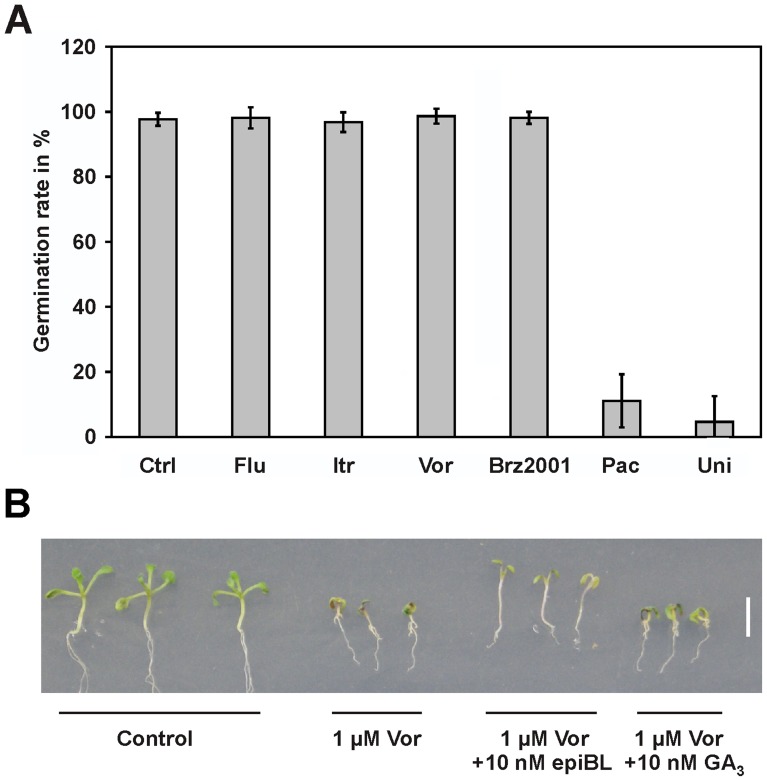
Voriconazole does not affect GA action. (**A**) In contrary to GA biosynthesis inhibitors voriconazole does not interfere with germination. 50 to 60 seeds were incubated on ATS media containing 25 µM of the indicated compounds, in long-day conditions at 24±2°C. Germination was assessed after 4 days. Data are the mean ± SD from 3 independent experiments. Paclobutrazole and unicozole treated samples are statistically highly significantly different from the other samples (ANOVA P-value <0.01%). (**B**) Application of GA does not rescue voriconazole induced growth inhibition. Seedling development in the presence of voriconazole, 24-epiBL or GA_3_. Plants were grown on ATS media supplemented with the indicated compounds for 12 days.

In an attempt to further investigate the mode of action by which voriconazole impacts on plant development we aimed to elucidate, if the growth defects observed in voriconazole-treated arabidopsis seedlings were caused by BR-deficiency or may also be due to a lack of GAs. An important difference in the action of these growth-promoting metabolites is that, in GA-deficient plants, germination is impaired, while BR and sterol mutants germinate normally [40], [41], [42], [43], [44], [45], [46]. Thus we decided to carry out germination experiments of arabidopsis seeds in the presence of selected triazoles (Figure 3A). We found that, in line with previous reports [47], [48], application of the GA biosynthesis inhibitors paclobutrazol and uniconazole reduced germination of arabidopsis seeds significantly (ANOVA P-values: <0.01). In contrast, the BR biosynthesis inhibitor Brz2001, as well as voriconazole, itraconazole and fluconazole did not impact on germination efficiency, indicating that GA biosynthesis was not affected substantially by the latter.

To further verify this result we conducted hypocotyl elongation assays with arabidopsis seedlings in which voriconazole was co-applied with 24-epiBL or the bioactive gibberellin GA_3_. The results illustrated in Figure 3B show that, whereas a co-application of 24-epiBL reverted voriconazole-suppressed hypocotyl elongation, GA_3_ did not achieve a rescue, providing additional support to the notion that voriconazole treatment does not significantly affect GA action. This result was confirmed when levels of GA_4_, the bioactive GA in arabidopsis, were measured and were found to be similar in voriconazole-treated seedlings as compared to untreated controls (GA_4_ levels of two independent samples: untreated 2.7, 3.0 ng/g Dw; treated with 3 µM voriconazole: 2.3, 2.1 ng/g Dw). Thus, there was evidence that voriconazole specifically affects BR action, likely by impacting on BR biosynthesis.

**Figure 4:**
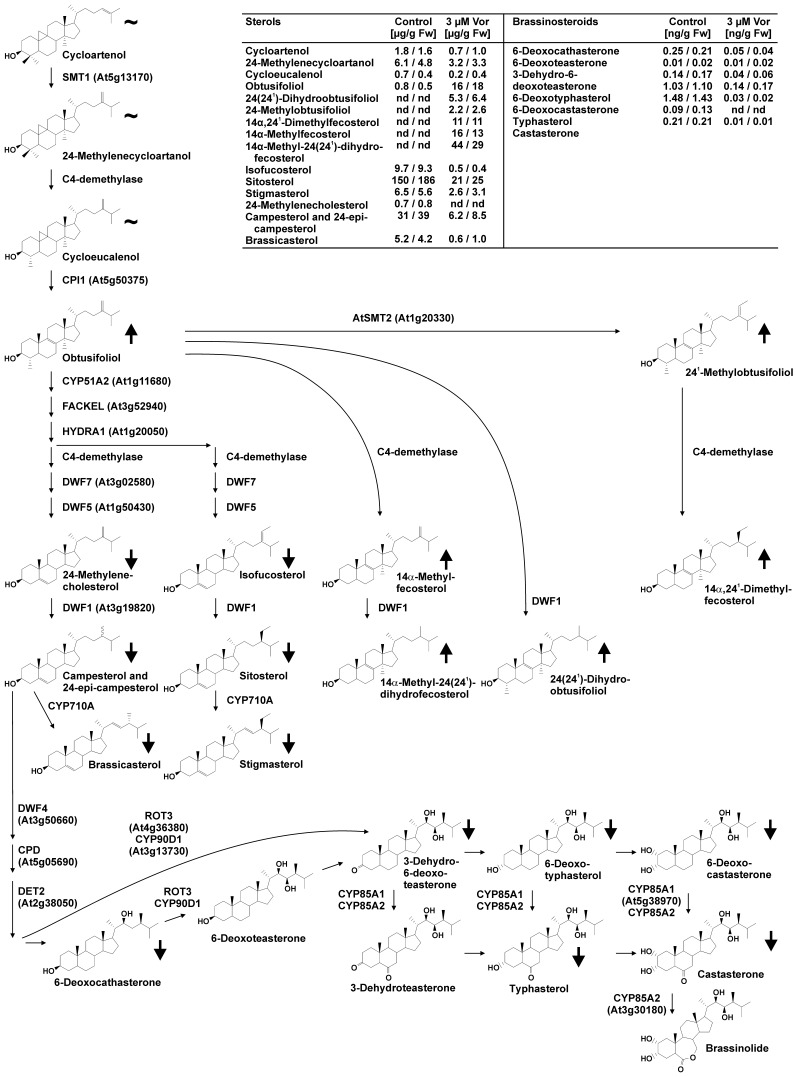
Voriconazole alters the sterol and BR profile of arabidopsis plants. Sterol and BR contents were quantified by GC-MS in two independent experiments in which arabidopsis seedlings grown either on media containing 3 µM voriconazole or on unsupplemented media for 10 d were compared. Sterol (µg/g fw) and BR levels (ng/g fw) are shown in the table. nd, not detected (below detection limit). For illustration changes are marked in the pathway, which was adapted from [46], [49], [50]. ↑, up; ↓, down; ∼, changes less than 2-fold. Biosynthetic enzymes of the cytochrome P450 family are in bold.

To conclusively assess whether BR production was impaired in voriconazole-treated plants, ten-day-old seedlings of arabidopsis grown on ATS plates containing 3 µM of voriconazole were analyzed for their BR contents using GC-MS. This analysis revealed that all BRs tested were strongly reduced in voriconazole-treated plants as compared to the untreated controls (Figure 4). The fact that the level of campesterol, a precursor of BR biosynthesis [49], [50], was markedly reduced in response to voriconazole treatment suggested that upstream sterol contents may also be affected. An analysis of sterol levels following voriconazole application revealed that the bulk sterols campesterol and sitosterol, as well as compounds derived of them, including the BRs, were significantly reduced in voriconazole-treated plants (Figure 4). 24-methylenecholesterol, the precursors of campesterol, isofucosterol and sitosterol were clearly reduced. In contrast, obtusifoliol and a number of unusual sterols derived of obtusifoliol were strongly increased in their levels (Figure 4).

In summary these results show that voriconazole acts as a strong inhibitor of sterol biosynthesis in arabidopsis and targets an enzymatic activity that catalyzes the conversion of obtusifoliol to 24-methylenecholesterol. A reduction of sterols was correlated with reduced levels of BRs and associated phenotypes, which could be rescued by BR application.

### Voriconazole Rapidly Enters Plant Tissues

**Figure 5:**
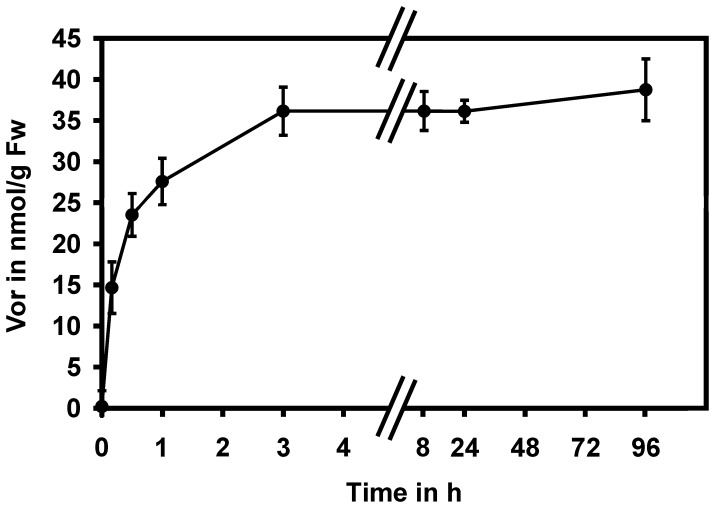
Voriconazole is rapidly incorporated. Arabidopsis seedlings were incubated for the indicated periods of time in ATS media supplemented with 25 µM voriconazole and the *in situ* contents of voriconazole were quantified by HPLC-DAD analysis. The means and standard deviations were calculated from four measurements.

The uptake rate is an important factor, which contributes to determining the *in vivo* potency of a chemical inhibitor. To investigate the tissue permeability of voriconazole in plants, seedlings of arabidopsis were incubated in liquid ATS media containing voriconazole. Samples were taken in a time-course manner and internalized voriconazole was quantified by HPLC-DAD analysis. As shown in Figure 5, 15 nmol/g Fw voriconazole were detected already 15 min after treatment. Within 3 hrs a plateau concentration of approximately 35 nmol/g Fw was reached and was then sustained for the rest of the experiment.

### Voriconazole Inhibits Growth of both Monocotyledonous and Dicotyledonous Crop Plants

**Figure 6:**
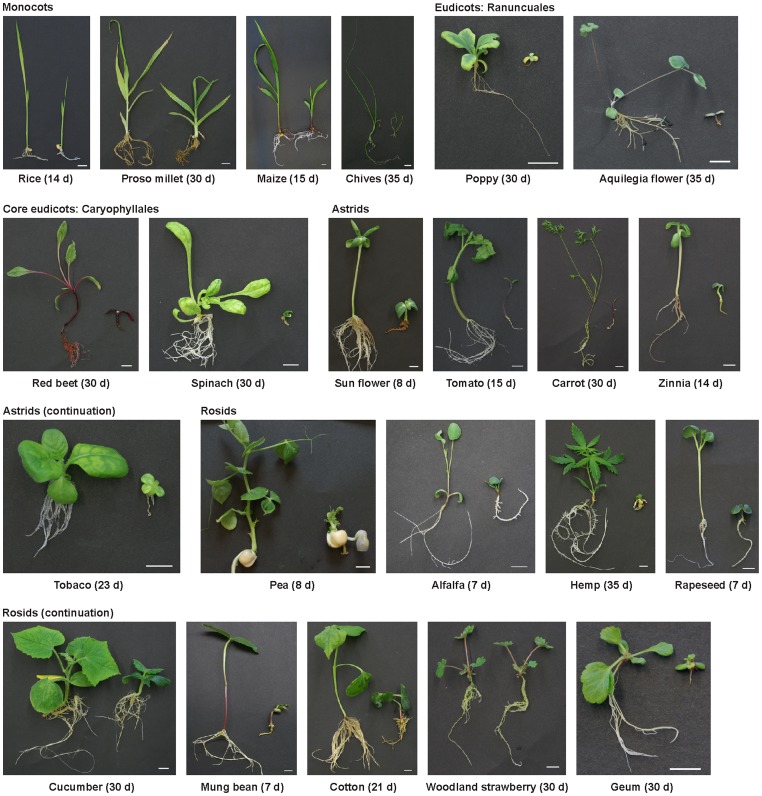
Voriconazole inhibits growth of monocotyledonous and dicotyledonous crop plants. Phenotypes of plants grown on control media (left) or media containing 1 µM voriconazole (right). The days after germination are indicated in brackets. The white scale bar represents 1 cm.

Our results showed that voriconazole acts as a highly potent inhibitor of BR action in arabidopsis. To determine if voriconazole had the ability to also inhibit growth of other plant species we tested its effects on a range of crops relevant for agriculture and horticulture in growth response assays carried out in ATS media containing 1 µM of the compound. The results of these assays showed that voriconazole acted on all monocotyledonous species analyzed, which were rice, proso millet, maize and chives (Figure 6). Phenotypes induced by voriconazole included inhibited leaf growth, reduced internode elongation and an impairment of root development. The strongest effects among monocots were observed for chives. The dicotyledonous crops investigated were poppy and aquilegia flower (Ranuncuales), red beet and spinach (Cayophyllales), tobacco, tomato, carrot, zinnia and sunflower (Astrids) and pea, alfalfa, hemp, cucumber, mung bean, rapeseed, cotton, woodland strawberry and geum (Rosids). All dicots tested were severely inhibited in their development by voriconazole application with the strongest effects observed for poppy, aquilegia flower, red beet, spinach and hemp, which arrested growth after cotyledon emergences. The only exception was the woodland strawberry *Fragaria vesca*, which showed normal shoot and root growth in the presence of 1 µM voriconazole. In contrast, the closely related species *Geum rivale* was highly sensitive to this drug (Figure 6).

To investigate whether the resistance of *F. vesca* was caused by reduced voriconazole uptake or rapid removal of the drug (by metabolism or modification) uptake studies were performed. Since a metabolite of *F. vesca* interfered with quantification of voriconazole by HPLC-DAD, we developed a HPLC-ESI-MS^2^-based assay. The results obtained for arabidopis using this method were comparable to those revealed by HPLC-DAD assays (Figure S4 and Figure S5). The kinetics of voriconazole uptake by *A. thaliana* and *F. vesca* were similar within the first 24 hrs. However, while a plateau concentration of approximately 25 nmol/g Fw was reached in arabidopsis, voriconazole levels increased in *F. vesca* to more than 50 nmol/g Fw. This result demonstrates that voriconazole resistance of *F. vesca* is caused by another mechanism than reduced uptake or a rapid removal of the drug.

### FvCYP51 Confers Voriconazole Resistance

A quantification of sterols in voriconazole-treated arabidopsis plants had shown a strong increase of obtusifoliol while the levels of intermediates further down-stream in sterol biosynthesis including 24-methylenecholesterol and isofucosterol were strongly decreased. Obtusifoliol is transformed to 24-methylenecholesterol by six steps of enzymatic modifications. Among them is the cytochrome P450 CYP51A2, which was a candidate for being a voriconazole target in plants since the drugs mode of action as a fungicide is an inhibition of CYP51s of yeast and filamentous fungi [51], [52].

**Figure 7:**
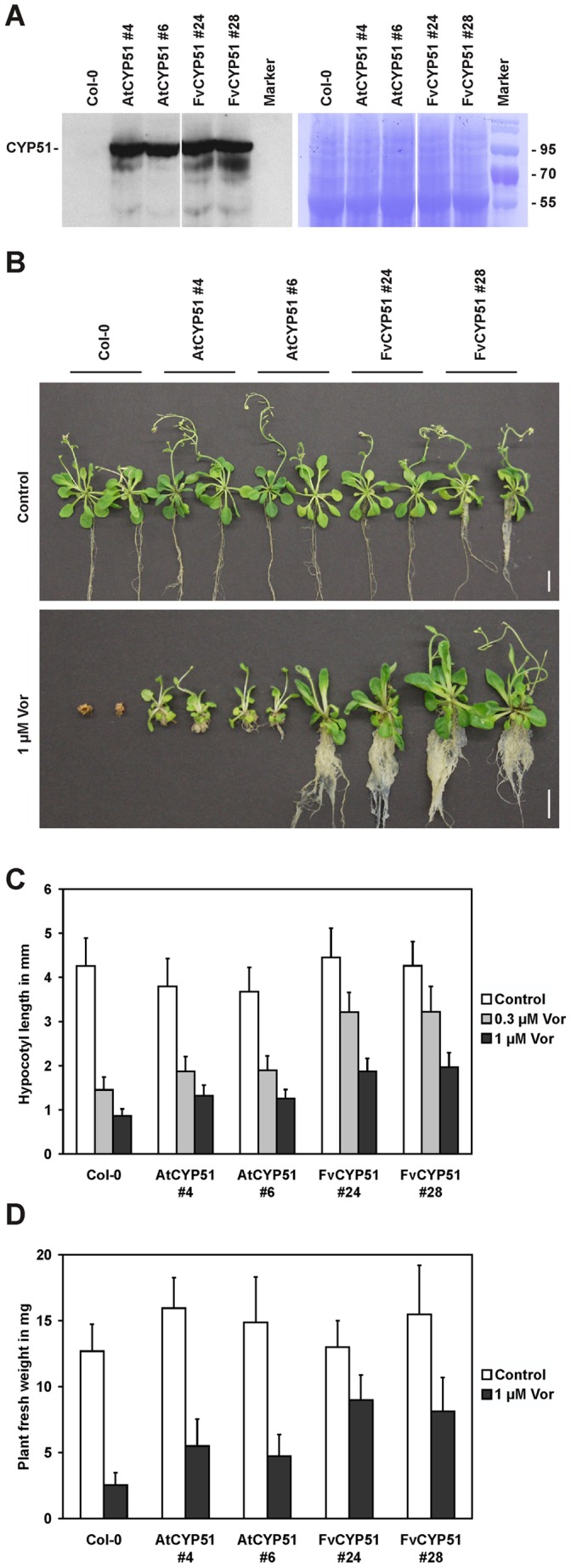
FvCYP51 confers voriconazole resistance. (**A**) Western blot analysis of lines expressing YFP-tagged versions of *AtCYP51A2* or *FvCYP51* under control of the 35S promoter (left panel). Staining of the membrane with coomassie brillant blue R250 shows loading of the samples. (**B**) Phenotype of *AtCYP51* and *FvCYP51* over-expressing lines and wild-type plants grown on ½ MS media or media complemeted with 1 µM voriconazole for 3 weeks. Two representative plants are shown for each line. Hypocotyl length (**C**) and biomass (**D**) of the same lines grown on ½ MS supplemented with the indicated concentration of voriconazole. Plants were grown under long day conditions for 10 day (**C**) or 2 weeks (**D**). Data are the mean ± SD of at least 20 plants measured.

Since we found clear difference in voriconazole resistance between *F. vesca* and *G. rivale*, we speculated that the predicted toxin target CYP51 might have evolved resistance in *F. vesca*. To investigate if CYP51 is a voriconazole target *in planta* and to determine if *FvCYP51* conferred resistance to the drug we constructed a YFP-tagged version under control of the strong, constitutive cauliflower mosaic virus 35S promoter and stably expressed it in arabidopsis plants. As a control we generated plants stably over-expressing a YFP-tagged version of arabidopsis *CYP51A2* (the only functional of two alleles in this species [46]). Lines expressing *AtCYP51* and *FvCYP51* to comparable levels were selected by western blot analysis (Figure 7A, left panel). Equal loading of the extracted protein was confirmed by staining the membrane with coomassie brilliant blue R250 (Figure 7A, right panel). The transgenic lines exhibited wild type-like phenotypes when grown in ½ MS media (Figure 7B, upper panel). In the presence of 1 µM voriconazole wild type Col-0 plants arrested growth shortly after germination and died within 3 weeks. Plants over-expressing *AtCYP51* showed a weak increase in tolerance, although they were severely impaired in growth. In contrast, plants over-expressing *FvCYP51* showed a high level of resistance to voriconazole, evidenced by hardly affected overall growth (Figure 7B, lower panel). Similarly, also hypocotyl elongation and biomass production was less affected in *FvCYP51* over-expressing plants than in the controls (Figure 7C and 7D).

## Discussion

In recent years significant progress has been made in our understanding of sterol biosynthesis and BR biosynthesis and signaling in plants, which was made possible largely by the use of molecular genetics in the model species *Arabidopsis thaliana* [3], [53], [10], [5]. However, sterol- and BR-deficient mutants are available for only a few plant species with agro-economical interest as classical mutant isolation is hampered in many crops by their polyploidy. Chemical inhibitors are an attractive option to circumvent this problem. Moreover, their application to different genetic backgrounds is possible without the need for time-consuming crossing and a transient application allows studying the impact of targeted pathways in certain tissues and developmental stages [19], [20].

Here we report that the triazole voriconazole is a potent inhibitor of plant growth and is active in a wide range of plant species. Voriconazole treatment of arabidopsis induced growth defects caused by BR deficiency as evidenced at the morphological level by impaired cell elongation, which was rescued by external application of BR. BR profiling of voriconazole-treated plants provided conclusive evidence that voriconazole impaired BR production, as the levels of all pathway intermediates measured were strongly reduced. A drastic reduction of the concentration of campesterol, a bulk sterol that serves as the biosynthetic precursor for BR biosynthesis [4], suggested that also sterol synthesis was inhibited by voriconazole treatment. Indeed, sterol levels in voriconazole-treated arabidopsis seedlings were significantly altered. The content of obtusifoliol was strongly increased as compared to control plants and a number of obtusifoliol-derived sterols, which are usually below the limit of detection in arabidopsis, were present in considerable amounts. In contrast, the levels of intermediates further down-stream in sterol biosynthesis including 24-methylenecholesterol and isofucosterol and its derivatives were strongly decreased following voriconazole application. Thus, the sterol profile suggested that an enzymatic activity responsible for the conversion of obtusifoliol to 24-methylenecholesterol and isofucosterol is affected by voriconazole application.

Obtusifoliol is converted to 24-methylenecholesterol by six steps of enzymatic modifications; five of the enzymes catalyzing these reactions in arabidopsis are known [5]. Among them is the cytochrome P450 CYP51A2, which was a likely candidate for being a voriconazole target. This is due to the fact that voriconazole is applied in human and veterinary medicine as an inhibitor of fungal CYP51s, which are sterol 14α-demethylases [51], [52]. CYP51 catalyzes demethylation of lanosterol in mammals and yeasts and demethylation of eburicol in filamentous fungi [54], [55] and is one of the most conserved P450s across the kingdoms [56]. CYP51A2 of arabidopsis converts obtusifoliol by C14-demethylation to 4α-methyl-5α-ergosta-8,14,24(24^1^)-trien-3β-ol [46]. A loss of CYP51A2 function results in alterations in sterol levels closely resembling those of voriconazole-treated plants: an accumulation of obtusifoliol and 14α-methyl-Δ8-sterols at the expense of campesterol and sitosterol [46]. Moreover, like voriconazole-treated plants, *CYP51A2* knock-out mutants (*cyp51a2*) showed reduced cell elongation leading to impaired shoot and root development [46]. In addition to these phenotypes, *cyp51a2* mutant plants display postembryonic seedling lethality, which is also characteristic for other mutants affected in the conversion of obtusifoliol to 24-methylene-cholesterol and isofucosterol (e.g. *smt1*, *fk/hyd2* or *hyd1*; [40], [42], [44]). Seedling lethality phenotypes of *fk*, *hyd1* and *smt1* have been attributed to a disturbed embryogenesis and are a maternal effect [40]. Although early embryogenesis is not strongly affected in *cyp51a2*, the mutant shows impaired seed development [46]. An application of voriconazole to healthy seedlings likely uncoupled the roles of CYP51A2 in the different phases of plant development and thereby revealed specific functions of CYP51A2 products in the seedling stage. Consistent with this hypothesis an application of voriconazole to flowers strongly affected seed development (Figure S3). In this context it is interesting to note that defects in seedling development induced by voriconazole treatment were rescued by BR application. Thus, in the seedling stage the bulk sterols isofucosterol, sitosterol and stigmasterol do not appear to be essential for development and CYP51 is primarily required to supply campesterol, as a precursor for BR production.

Our results show that voriconazole acts to inhibit BR-dependent sterol biosynthesis and more specifically, obtusifoliol 14α-demethylase activity *in planta*. Compounds acting on this activity have been identified previously [29], [31]. However, some of these inhibitors are not specifically targeting sterol production as they additionally inhibit the cytochrome P450-mediated oxidation steps of *ent*-kaurene to *ent*-kaurenoic acid and thereby also reduce GA biosynthesis [29]. As opposed to known GA biosynthesis inhibitors such as paclobutrazol and uniconazole, voriconazole did not reduce the germination efficiency of arabidopsis seeds. Moreover, voriconazole-induced growth defects were not rescued by GA application and GA_4_ levels were not significantly reduced, showing that growth inhibition was not due to GA_4_ deficiency. Some triazole fungicides with structural similarities to voriconazole (Table S1), for instance propiconazole [24], [25] and ketoconazole [57], were previously shown to cause BR-deficient phenotypes and were speculated to inhibit BR biosynthesis. However, their molecular targets have remained elusive. In light of the result that the phenotype of plants treated with sterol and BR biosynthesis inhibitors is almost identical, it should be considered that these compounds may also impact on CYP51 action.

Since we found a striking difference in voriconazole resistance between *F. vesca* and *G. rivale* and since *FvCYP51*, when over-expressed in arabidopsis, conferred resistance to the drug we speculated that sequence alterations in CYP51 between the two evolutionary closely related species might exist. To assess this we cloned the *CYP51s* of both plants and aligned their sequences together with published CYP51 sequences from other identified sensitive genera (arabidopsis, tobacco and tomato; Figure S5). This showed that only few amino acids were conserved in CYP51s of voriconazole-sensitive plants but were substituted in *F. vesca*: namely L18, R247, C248, Y259, D273 and T395 (Figure S5). In yeast and filamentous fungi where CYP51 mutations conferring triazole insensitivity have been extensively studied, none of these amino acids has as yet been found to confer resistance (Figure S5) suggesting that voriconazole target sites in plant CYP51s may differ from those of yeast and fungi. Alternatively, but not mutually exclusive to the notion of an altered target, FvCYP51 may have an increased catalytic activity towards its substrate.

In contrast to yeast and filamentous fungi, where accumulation of 14α-methylated sterols is an important mode of triazole toxicity [58], [59], we here show that voriconazole acts by inhibiting steroid hormone action in plants. Since this ability is naturally lost in *F*. *vesca* and considering that CYP51 inhibitors are studied as potential herbicides [60], it should be noted that targeted weed species could evolve resistance. An over-expression of *FvCYP51* in arabidopsis, a heterologous plant system, conferred a high level of inhibitor insensitivity; thus an application of *FvCYP51* over-expression as a tool to confer triazole resistance is conceivable.

### Conclusion

In conclusion our work identifies voriconazole as a novel inhibitor for dissecting the importance of CYP51 function and BR-dependent sterol biosynthesis in different tissue types and stages of plant development and for analyzing their function in a wide range of plant species for which no mutants are available. In contrast to known inhibitors targeting sterol 14α-demethylase activity voriconazole does not interfere with GA_4_ production and is commercially available, which will facilitate its use in basic research. Since modes of action, which confer voriconazole target site resistance may not be conserved between biological kingdoms, future research elucidating voriconazole action and resistance in plants is warranted.

## Methods

### Chemicals

Bifonazole, fluconazole, itraconazole, thiabendazole and uniconazole were purchased from LKT Laboratories (St. Paul, MN, USA). Econazole, sulconazole, 2-acetonaphthone and 24-epi-BL were obtained from Sigma (St. Louis, MO, USA). GA_3_ and paclobutrazol were purchased from Duchefa (Haarlem, The Netherlands). Brz2001 was a kind gift from Tadao Asami and Shigeo Yoshida. Voriconazole was obtained from Pfizer (New York, NY, USA). All compounds were dissolved in DMSO. Note: voriconazole is commercially available as “Vfend 200 mg”. This preparation contains β-cyclodextrin-sulfobutylether sodium salt, which renders voriconazole water soluble, but does not interfere with its biological activity in plants. However, for this study pure voriconazole was used.

### Plant Material and Growth Conditions

The plant species and cultivars used in this study were: *Allium schoenoprasum* cv. Staro (chives), *Aquilegia caerulea* (aquilegia flower), *Arabidopsis thaliana* ecotype Col-0 (arabidopsis), *Beta vulgaris* cv. Rote Kugel 2 (red beet), *Brassica napus* cv. Westar (rapeseed), *Cannabis sativa L.* (hemp), *Cucumis sativus* cv. Naf/Fanto (cucumber), *Daucus carota* cv. Rothild (carrot), *Fragaria vesca* cv. Rügen (woodland strawberry), *Geum rivale* cv. Goldball, *Gossypium herbaceum* (cotton), *Helianthus annuus* cv. Riesenrad (sunflower), *Lepidium sativum* (cress), *Solanum lycopersicum* cv. Harzfeuer (tomato), *Medicago sativa* (alfalfa), *Nicotiana tabacum* cv. SR1 (tobacco), *Oryza sativa* cv. Nipponbare (rice), *Panicum miliaceum* (proso millet), *Papaver orientale* (poppy), *Pisum sativum* cv. Sugar Bon (pea), *Spinacia oleracea* cv. Matador (spinach), *Vigna radiata* (mung bean), *Zea mays* cv. Tasty Gold (maize) and *Zinnia elegans* (zinnia).

For growth response assays on sterile media arabidopsis and cress seeds were surface-sterilized with chlorine gas as described previously [61] and were then plated on solid ATS media [62] containing 1% sucrose as well as the described compounds. After stratification at 4°C for 2 days the seeds were placed in a growth chamber at 24±2°C and incubated in long-day growth conditions (16 hrs 80 µmol•m^−2^•s^−1^ cool white light/8 hrs dark). For germination experiments 50 to 60 arabidopsis seeds were plated on solid ATS media supplemented with the indicated compounds. The plates were then stratified for 2 days at 4°C and shifted to 24±2°C and long-day conditions. Germination, defined as emergence of the cotyledons, was determined after 7 days using a Zeiss 2000-C stereomicroscope (Carl Zeiss, Inc., Jena, Germany) with 40×magnification.

Chive seeds were surface-sterilized with chlorine gas as described above. Seeds of all other plant species used were sterilized using an initial washing step with 1% soap, which was followed by a treatment with 70% ethanol for 3 min and a treatment with diluted bleach (containing 0.3% sodium hypochlorite) for 5 min. The seeds were then rinsed 4 times with sterile water and were plated on ATS plates supplemented with the indicated compound. Seeds of cotton were treated with concentrated sulfuric acid (15 min) and washed 4 times with water prior to surface sterilization.

### Generation of Transgenic Lines

The *FvCYP51* gene of *F. vesca* was amplified using degenerated primers designed for the most conserved region of plant CYP51s. The obtained amplicon was sequenced and the obtained sequence used to design primers for inverse PCR, which allowed to obtain the full 5′ UTR. In a second round of inverse PCR also the remaining parts could be sequenced. Subsequently the whole genomic fragment was cloned into the vector pGWR8 [12]. For *AtCYP51A2* the sequence available in the TAIR database was used to design primers and the obtained amplicon was cloned into pGWR8. Arabidopsis Col-0 plants were transformed with these constructs using the floral dip method [61].

### Permeability Assays

For permeability assays *A. thaliana* and *F. vesca* plants were incubated in liquid ATS media containing 25 µM voriconazole for the indicated amounts of time, rinsed with distilled water, dried on filter paper and frozen in liquid nitrogen. For the analysis plant material was ground to a fine powder and 1 ml of extraction buffer (10 mM sodium formiate, pH 4.0, 40% methanol for HPLC-DAD; 40% methanol for HPLC-ESI-MS^2^) was added to 100 mg of each sample. The mixtures were spiked with 50 µl stock solution of either 50 µM 2-acetonaphthone for HPLC-DAD or 2.58 mM MUG (4-methylumbeliferyl-β-D-glucuronide) for HPLC-ESI-MS^2^ as internal standard. Extraction was performed at 60°C for 1 hr in an ultrasonic bath with occasional shaking. The extracts were centrifuged 2 times for 5 min at 15,000 g and the cleared supernatants were then directly used for HPLC-DAD or HPLC-ESI-MS^2^ analysis.

The HPLC-DAD system was comprised of a Dionex P680 pump, an ASI-100 autosampler and a PDA-100 diode array detector (DAD). The system was equipped with a Macherey-Nagel 125×4 mm Nucleosil 100-5 C_18_ HD column preceded by a Macherey-Nagel 8×4 mm Nucleosil 100-5 C_18_ pre-column. A constant flow rate of 1 ml/min was maintained with a gradient of solvent A (60 mM formic acid set to pH 4.0 with NaOH; 8.5% acetonitrile) and solvent B (pure methanol). Elution began with an isocratic flow of solvent A for 1 min. The concentration of solvent B was then raised linearly to 100% in 19 min and kept isocratic for another 2 min prior to reducing it to 0% within 1 min. The column was equilibrated for 5 min with solvent A before injection of the next sample. The absorbance was recorded in the range of 220 nm to 500 nm with 1 nm intervals. Compounds were identified by their retention times and their UV spectra. For quantification the absorbances at 258 nm (for voriconazole) and at 285 nm (for 2-acetonaphthone) with bandwidths of 10 nm were used.

For HPLC-ESI-MS^2^ analysis a Bruker Daltonics esquire 3000^plus^ ion trap mass spectrometer (Bruker Daltonics, Bremen, Germany) connected to an Agilent 1100 HPLC system (Agilent Technologies) equipped with a quaternary pump and a diode array detector was used. Components were separated with a Phenomenex Luna C-18 column (150 mm×2.0 mm, particle size 5 ***µ***m; Phenomenex, Aschaffenburg, Germany) that was held at 28°C. HPLC was performed with the following binary gradient system: solvent A, water with 0.1% formic acid and solvent B, 100% methanol with 0.1% formic acid. The gradient program was as follows: 0–30 min, 100% A to 50% A/50% B; 30–35 min, 50% A/50% B to 100% B, hold for 15 min; 100% B to 100% A, in 5 min, then hold for 10 min. The flow rate was 0.2 ml/min. The ionization parameters were as follows: the voltage of the capillary was 4000 V and the end plate was set to −500 V. The capillary exit was 121 V and the Octopole RF amplitude 150 Vpp. The temperature of the dry gas (N_2_) was 330°C at a flow of 9 l/min. Tandem MS was carried out using helium as the collision gas (3.56·10^−6^ mbar) with 1 V collision voltage. Spectra were acquired in positive ionization mode and target ions were fragmented in manual MS^n^ mode. The most prominent product ions were used for quantification (all isons were detected in the positive mode; Vor: *m/z* 127, 224 and 281; MUG: *m/z* 177).

### Measurement of Sterol, BR and GA Level

Quantification of sterols, BRs and GA_4_ was carried out according to methods described previously [63], [64], [65].

### Western Blot Analysis

Samples (100 mg each) of ten-day-old seedlings of the generated *35S:AtCYP51-YFP* and *35S:FvCYP51-YFP* lines were shock-frozen and homogenized in liquid nitrogen, then 300 µl extraction buffer (67 mM TRIS pH 6.8, 133 mM DTT, 2.7% SDS, 13% glycerol, 0.01% bromophenol blue) were added and immediately incubated at 95°C for 2 min. After centrifugation 10 µl of the extracts were separated by SDS-PAGE (10% gel) and blotted onto a polyvinylidene difluoride membrane (Millipore, Billerica, MA). After blocking with a solution of 5% skim milk powder in TBS-T (150 mM NaCl, 10 mM TRIS/HCl pH = 8.0, 0.1% Tween 20) the membrane was probed with an anti-GFP antibody (Roche, Basel, Switzerland). Alkaline phosphatase-conjugated goat anti-mouse Fab specific fragment (Sigma-Aldrich, St. Louis, MO, USA) was employed as secondary antibody. For detection the CDP-Star™ detection reagent (GE Healthcare, Buckinghamshire, UK) was used. To verify equal loading the membrane was subsequently stained with coomassie brilliant blue R-250 dissolved in water/2-propanol/acetic acid = 650/250/100 and finally destained with water/2-propanol/acetic acid = 750/150/100.

### Electron Microscopy of Hypocotyls

Hypocotyls were fixed on a metal support rack with Tissue-Tek (Sakura Finetek, Torrance, CA) and shock frozen in liquid nitrogen. Epidermal hypocotyl cells were then visualized with a Hitachi T-1000 scanning electron microscope (Hitachi High-Tech, Tokyo, Japan).

## Supporting Information

Figure S1 Fluconazole treatment induces BR-deficient like phenotypes. The phenotype of arabidopsis plants treated with fluconazole is shown.
(PDF)

Figure S2 Voriconazole reduces cell elongation. Electron micrographs of zinnia and cress hypocotyls with and without voriconazole treatment are shown.
(PDF)

Figure S3 Voriconazole affects seed development. The phenotype of arabidopsis siliques from plants with and without voriconazole treatment are shown.
(PDF)

Figure S4 Uptake of voriconazole by *A. thaliana and F. vesca*. The uptake of voriconazole by *A. thaliana* and *F. vesca* is compared.
(PDF)

Figure S5 Alignme,nt of CYP51 amino acid sequences. The data provide an alignmet of the CYP51 protein sequences from *Fragaria vesca* (woodland strawberry), *Geum rivale*, *Arabidopsis thaliana*, *Solanum lycopersicum* (tomato) and *Nicotiana tabacum* (tobacco).
(PDF)

Table S1 Compounds interfering with sterol and BR biosynthesis. The structures of compounds inhibiting sterol or BR biosynthesis are compared.
(PDF)
