# Supplementary material for: Genetic Variation in Plant CYP51s Confers Resistance against Voriconazole, a Novel Inhibitor of Brassinosteroid-Dependent Sterol Biosynthesis
Source: PLoS One. 2013 Jan 15;8(1):e53650. doi: 10.1371/journal.pone.0053650 (PMC3546049; doi:10.1371/journal.pone.0053650)
Supplement: Figure S1 — Fluconazole treatment induces BR-deficient like phenotypes. The phenotype of arabidopsis plants treated with fluconazole is shown. (PDF) [file pone.0053650.s001.pdf]

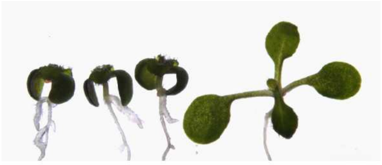

Figure S1. Fluconazole treatment induces BR-deficient like phenotypes. Arabidopsis plants were grown for 7 d on ATS plates containing 25 µM fluconazole (left) or on unsupplemented plates as controls (right).
