# Supplementary material for: Genetic Variation in Plant CYP51s Confers Resistance against Voriconazole, a Novel Inhibitor of Brassinosteroid-Dependent Sterol Biosynthesis
Source: PLoS One. 2013 Jan 15;8(1):e53650. doi: 10.1371/journal.pone.0053650 (PMC3546049; doi:10.1371/journal.pone.0053650)
Supplement: Figure S2 — Voriconazole reduces cell elongation. Electron micrographs of zinnia and cress hypocotyls with and without voriconazole treatment are shown. (PDF) [file pone.0053650.s002.pdf]

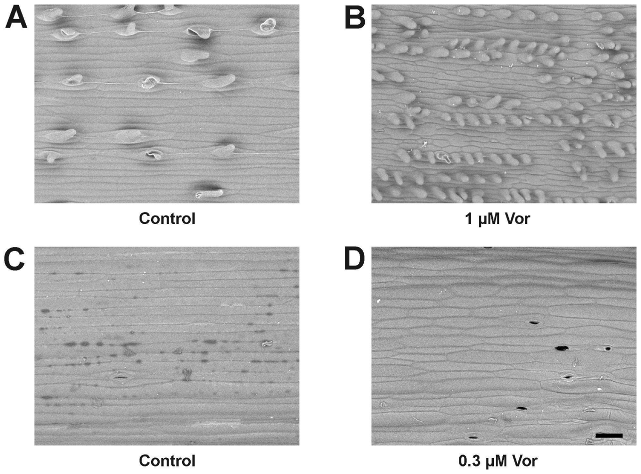

Figure S2. Voriconazole reduces cell elongation. Zinnia (A and B) and cress (C and D) plants were grown on control media or on plates supplemented with the indicated concentration of voriconazole for 10 and 5 days, respectively. Pictures of epidermal hypocotyl cells were taken by electron microscopy. The bar represents 50 µm. All pictures were taken with the same magnification.
