# Supplementary material for: Genetic Variation in Plant CYP51s Confers Resistance against Voriconazole, a Novel Inhibitor of Brassinosteroid-Dependent Sterol Biosynthesis
Source: PLoS One. 2013 Jan 15;8(1):e53650. doi: 10.1371/journal.pone.0053650 (PMC3546049; doi:10.1371/journal.pone.0053650)
Supplement: Figure S3 — Voriconazole affects seed development. The phenotype of arabidopsis siliques from plants with and without voriconazole treatment are shown. (PDF) [file pone.0053650.s003.pdf]

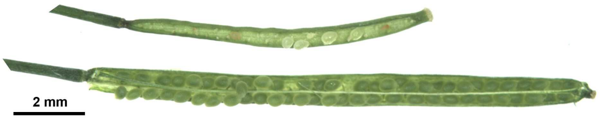


**Figure S3. Voriconazole affects seed development.** Arabidopsis plants were sprayed for 4 weeks with either 10 µM Vor (upper silique) or, as a control, with water (lower silique) 5 times a week prior microscopic observation.
