# Supplementary material for: Genetic Variation in Plant CYP51s Confers Resistance against Voriconazole, a Novel Inhibitor of Brassinosteroid-Dependent Sterol Biosynthesis
Source: PLoS One. 2013 Jan 15;8(1):e53650. doi: 10.1371/journal.pone.0053650 (PMC3546049; doi:10.1371/journal.pone.0053650)
Supplement: Figure S4 — Uptake of voriconazole by A. thaliana and F. vesca. The uptake of voriconazole by A. thaliana and F. vesca is compared. (PDF) [file pone.0053650.s004.pdf]

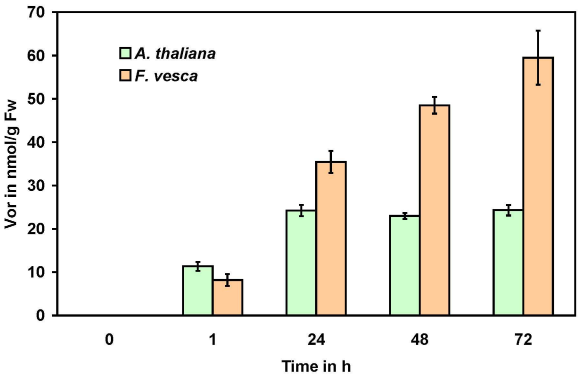

Figure S4. Uptake of voriconazole by *A. thaliana and F. vesca*. Three-week-old *A. thaliana* and *F. vesca* plants were transferred to liquid ATS media containing 25 µM voriconazole and incubated for the indicated time. Subsequently samples were taken and analzed by HPLC-ESI-MS². The average and standard deviation of three biological replicates is shown.
