# Supplementary material for: Genetic Variation in Plant CYP51s Confers Resistance against Voriconazole, a Novel Inhibitor of Brassinosteroid-Dependent Sterol Biosynthesis
Source: PLoS One. 2013 Jan 15;8(1):e53650. doi: 10.1371/journal.pone.0053650 (PMC3546049; doi:10.1371/journal.pone.0053650)
Supplement: Table S1 — Compounds interfering with sterol and BR biosynthesis. The structures of compounds inhibiting sterol or BR biosynthesis are compared. (PDF) [file pone.0053650.s006.pdf]

Table S1: Compounds interfering with sterol and BR biosynthesis

	Compound	Structure	Evidence [Reference]	Availability
Compounds targeting CYP51	Voriconazol	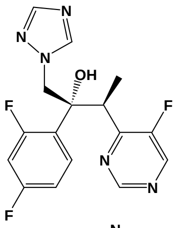	Sterol, BR and GA profile, expression of a resistant CYP51 variant, hypocotyl elongation and rescue assays [This study]	Pfizer (Vfend)
	LAB 170250F	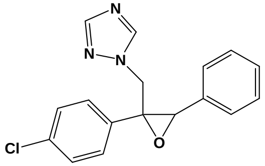	Sterol profile, expression of a resistant CYP51 variant [3]	Not commercially available
Compounds targeting DWF4	Brassinazole	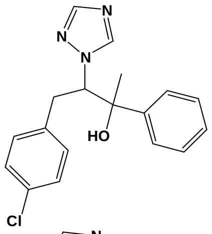	Hypocotyl elongation and rescue assays [1], binding of Brassinazole to DWF4 [2]	TCI (no. B2829)
	Brz220	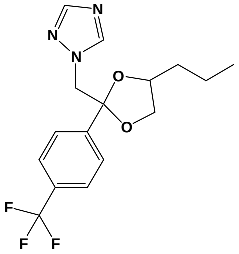	Hypocotyl elongation and rescue assays [6], binding of Brz220 to DWF4 [8]	Not commercially available

Table S1: Compounds interfering with sterol and BR biosynthesis (continuation)

	Compound	Structure	Evidence [Reference]	Availability
Compounds with unknown targets	Brz2001	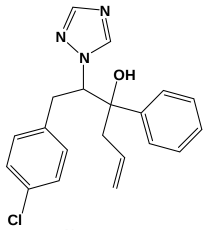	Hypocotyl elongation and rescue assays with 24-epiBL and GA_3. The structural similarity to brassinazole suggests DWF4 as a pontential target [7]	Not commercially available
	Propiconazole	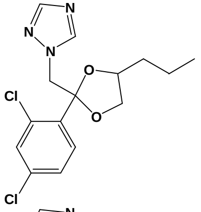	Hypocotyl elongation and rescue assays with 24-epiBL and GA_3 [6,4]. DWF4 might be the molecular target of propiconazole [4]	Santa Cruz Biotchnology (sc-250786)<br>Sigma-Aldrich (no. 45642)<br>Toronto Research Chemicals (no. P770100)
	Ketoconazole	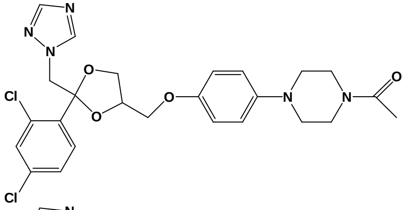	Hypocotyl elongation and rescue assays with 24-epiBL and GA_3 [5]	Enzo Life Sciences (no. BML-EI107)<br>LKT Laboratories (no. K1676)<br>Sigma-Aldrich (no. 45642)
	Itraconazole	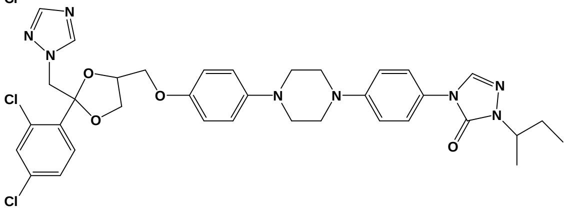	Hypocotyl elongation assays [This study]	Biovision (no. 1987-50)<br>LKT Laboratories (no. I7870)<br>Sigma-Aldrich (no. I6657)

References

1. Asami T, Min YK, Nagata N, Yamagishi K, Takatsuto S, et al. (2000) Characterization of brassinazole, a triazole-type brassinosteroid biosynthesis inhibitor. Plant Physiol 123: 93-100.
2. Asami T, Mizutani M, Fujioka S, Goda H, Min YK, et al. (2001) Selective interaction of triazole derivatives with DWF4, a cytochrome P450 monooxygenase of the brassinosteroid biosynthetic pathway, correlates with brassinosteroid deficiency in planta. J Biol Chem 276: 25687-25691.
3. Grausem B, Chaubet N, Gigot C, Loper JC, Benveniste P (1995) Functional expression of *Saccharomyces cerevisiae* CYP51A1 encoding lanosterol-14-demethylase in tobacco results in bypass of endogenous sterol biosynthetic pathway and resistance to an obtusifoliol-14-demethylase herbicide inhibitor. Plant J 7: 761-770.
4. Hartwig T, Corvalan C, Best NB, Budka JS, Zhu JY, et al. (2012) Propiconazole is a specific and accessible brassinosteroid (BR) biosynthesis inhibitor for arabidopsis and maize. PLoS One 7: e36625.
5. Oh K, Yamada K, Asami T, Yoshizawa Y (2012) Synthesis of novel brassinosteroid biosynthesis inhibitors based on the ketoconazole scaffold. Bioorg Med Chem Lett 22: 1625-1628.
6. Sekimata K, Han SY, Yoneyama K, Takeuchi Y, Yoshida S, et al. (2002) A specific and potent inhibitor of brassinosteroid biosynthesis possessing a dioxolane ring. J Agric Food Chem 50: 3486-3490.
7. Sekimata K, Kimura T, Kaneko I, Nakano T, Yoneyama K, et al. (2001) A specific brassinosteroid biosynthesis inhibitor, Brz2001: evaluation of its effects on Arabidopsis, cress, tobacco, and rice. Planta 213: 716-721.
8. Sekimata K, Ohnishi T, Mizutani M, Todoroki Y, Han SY, et al. (2008) Brz220 interacts with DWF4, a cytochrome P450 monooxygenase in brassinosteroid biosynthesis, and exerts biological activity. Biosci Biotechnol Biochem 72: 7-12.
